# A Proposed Theoretical Model for Sustainable and Safe Commensality among Older Adults

**DOI:** 10.3390/ijerph18031172

**Published:** 2021-01-28

**Authors:** Ingela Marklinder, Margaretha Nydahl

**Affiliations:** Department of Food Studies, Nutrition and Dietetics, Uppsala University, 751 22 Uppsala, Sweden; Margaretha.Nydahl@ikv.uu.se

**Keywords:** older adults, food classes, food safety, food safety trust, organized networking, sustainable commensality

## Abstract

Eating together at the same table, i.e., commensality, is an old phenomenon among humans. Today, there is a relatively high number of people living in single households eating most meals on their own. Among adults aged 65+ years, both malnutrition and non-communicable diseases are common. These circumstances, as well as foodborne illnesses, cause health problems for the individual, as well as high societal costs. In older adults, several external factors might impact on commensality, such as living arrangements, health status, and cooking competence. Improved knowledge regarding healthy eating and food handling may improve attitudes and behaviors in relation to food safety and dietary intake. Further, commensality has been shown to influence dietary intake in multiple ways. Community-organized activities, e.g., Food Classes for Older Adults (FCOA), may lead to sustainable commensality. Participating in health-promoting activities can contribute to improved health outcomes and improved social interaction among older adults. The objective of this study was to propose a theoretical model to inspire and create networks for sustainable commensality among older adults. The model could serve as a conceptual framework when implementing FCOA in communities and research. Outcomes could be measured by investigating the frequency of commensality, health effects, and well-being.

## 1. Introduction

A case study: “A single woman, aged 65+ years, was standing in the supermarket reading the label on a readymade asparagus soup when a man, also living alone, turned up. Actually, they were neighbors. They started to discuss the ingredient list. What is E451? How does this soup taste? The man spontaneously invited the woman to eat at his home. Would it be OK to accept the invitation? Would he wash his hands before cooking? What if she got food poisoning? Suddenly, an immunocompromised friend of the woman joined them. In the end, she accepted the invitation too, provided that she could ask her best friend to join them.

On the man’s way home, some questions popped up in his head: Can I make asparagus soup? I have no cooking experience, so will it be tasty enough? Will it be too expensive? What kind of bread goes with that kind of soup? Will they expect a nicely set table and do we need a dessert?”

A major challenge to public health in European countries is maintaining the health and quality of life in the aging population. Limited research has been aimed at food knowledge interventions including cooking and food safety to prevent or delay ill health for this population group. The objective of this study was to propose a theoretical model to inspire and create networks for sustainable commensality among older adults.

**Figure 1:**
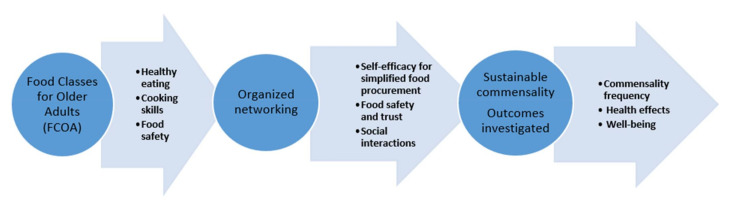
A theoretical model for sustainable commensality among older adults. An organized network is initiated by the practitioners at the Food Classes for Older Adults (FCOA), to be continued also after the activity.

A theoretical model for sustainable commensality among older adults is presented in Figure 1. The model includes the concept “Food Classes for Older Adults” (FCOA), which involves providing knowledge on nutrition, cooking, and food safety, as well as organized networking. The commensality frequency, health effects and well-being among the older adults are proposed to be investigated. The model can serve as a conceptual framework when implementing FCOA in communities and research.

### 1.1. Definitions of Commensality

Eating together at a table is an old phenomenon among human beings. The sociologists Durkheim, Simmel, and Weber were among the first scientists reflecting on the term *commensality*. The concept was divided in three different dimensions: *interactional* (an act of communication), *normative* (staging and control of norms by diners) and *symbolic* (the meanings attached to eating together) [1]. Fischler defined commensality as: “Eating together at the same table” [2]. Chee-Beng discussed the origin of the word commensality, stating that commensal means “one who eats at the same table” deriving from the Latin words *com* (together) and *mensalis* (of the table) [3]. Results from several studies have revealed that a deeper understanding of the topic and more data are necessary [4,5,6,7]. In this article, we use the broad definition “Eating together at the same table.”

### 1.2. Identifying the Problem

*Commensality* is an old phenomenon, but in modern times, 40 percent of the households in Sweden are single households [8]. This increases the number of people eating meals on their own. Among older adults—i.e., those aged 65+ years—there are two common major health problems: non-communicable diseases and malnutrition [9,10]. In addition to that, there are foodborne illnesses [11]. These conditions not only cause health problems for the individual, but also societal costs [12,13].

Commensality has been shown to influence dietary intake, but also has wider implications. Several external factors have an impact on commensality and its relation to health, such as the social situation, the timing of the meal, and the amount of food provided [5]. In a review by Vesnaver and Keller [4], it was concluded that social activity is an important factor for improved diet, but also that more studies, especially longitudinal ones, were required to understand how various social factors influence diet. Social isolation is an important risk factor predicting poor health in older adults [4]. The psychosocial aspects of aging are affected by the COVID-19 pandemic in multiple ways [14]. The restrictions put in place will obviously be an obstacle for any strategy to increase commensality among older people, who are specifically classified in the vulnerable group. However, Dubey et al. [14] have stated that the coronavirus “made us realize that the greatest assets of mankind are health, peace, love, solidarity, ingenuity, and knowledge”.

According to Herman, “The social facilitation of eating refers to increases in food intake when people eat together as compared to when they eat alone” [5]. In the same review by Herman, two main paradigms were defined: *audience effects* and *co-action effects*. *Audience effects* refer to when people behave in front of a passive audience, whereas *co-action effects* refer to when people behave alongside others engaged in the same behavior [15,16]. Herman suggested some situational effects on meal intake and analyzed to what extent the presence and behaviors of others inhibited or disinhibited eating. It was found that eating with family and friends led to greater intake than eating with strangers did [5].

Eating together in a private setting has also been shown to affect the confidence people feel in procurement of food for cooking and food safety routines. In a private household, you are not obliged to follow the routines for food hygiene pursuant to food-related legislation. Consumers in general have varied knowledge regarding cold food storage [17,18,19].

Relatively few studies have been performed regarding the domestic situations and mealtime experiences among community-dwelling older adults [20]. Living alone in older age has been shown to be associated with reduced motivation to cook and eat regular meals [21]. Retired people may face irregular meal patterns, after having spent their professional lives on a fixed schedule with regular mealtimes.

A study was performed on 297 community-dwelling individuals (men and women) aged 80+ years who reported poor appetite [22]. Almost all participants (99%) ate their main meal at home and men had company during meals significantly more often than women. Social aspects of eating in relation to nutrient intake (*n* = 770) were studied by Pachucki et al. [6]. Their study showed no significant association between the frequency of eating meals with others and nutrient intake for either men or women [6].

### 1.3. Food Safety and Commensality

A source of food safety knowledge is often family and friends, but food safety education increases knowledge [23]. In Europe, it has been estimated that one third of all foodborne outbreaks occur in private households [24,25]. One common cause of food poisoning, Shiga toxin-producing *E. coli* bacteria, is classified as a serious risk to public health due to a low infectious dose, with older adults being especially sensitive to complications such as hemolytic-uremic syndrome [26]. Another common bacterium in cases of cross-contamination is *Campylobacter spp.*, which creates a health risk for instance when preparing raw chicken; it also has a low infectious dose. *Listeria monocytogenes* could contribute to serious foodborne infection as it grows also at low temperatures. 

Though European food companies have to observe the legislative requirements related to food and are regularly reviewed by government inspectors, consumers are not subject to supervision in their food handling. Several studies on consumer behavior in private households indicate that food safety knowledge, attitude and practices are crucial [17,18,27,28,29,30,31]. Data from the EU project CHANCE (2007–2009) revealed several factors contributing to knowledge gaps among older adults in terms of food handling and hygiene [31,32]. One third of 251 consumers revealed knowledge gaps relating to storage temperature for certain food items. Twenty percent of the elderly often put raw minced meat in their mouth to taste seasoning, without reflecting on pathogenic bacteria. There was no significant relation between the fear of food poisoning and tasting raw minced meat, frequently changing their dishcloth, or cooling food properly. Some elderly people, who had grown up in the countryside and experienced slaughter and old-fashioned preservation techniques, argued that their routines went back many years and had not had any negative consequences [31]. Typically, they considered themselves fully capable of handling foodstuffs. The attitude that one already knows everything may hamper one’s receptiveness to relevant new health information [32,33]. Storage temperature and storage time are important factors influencing the risks for food poisoning. A study performed on 100 older adults (60+ years) investigated cognition and behaviors in relation to domestic food handling and storage practices that may increase the risks associated with *L. monocytogenes* [34]. Although the majority (79%) had positive attitudes toward refrigeration, 84% were unaware of recommended temperatures and 65% self-reported ‘‘never’’ checking their refrigerator temperature. A majority (84%) reported that they consumed readymade foods after the use-by date, which may increase risks associated with *L. monocytogenes*. The results from these two studies can be interpreted as signs of knowledge gaps, indicating a need for improved health communication.

### 1.4. Hospitality and Commensality

Inviting a friend to share a meal may have certain benefits due to positive effects related to commensality. In an earlier study among older woman by Sidenvall et al. [21], the whole procedure of preparing a meal could be seen as a gift. Four phases were identified: figuring out what to serve, cooking with fresh ingredients, presenting the gift in a beautiful manner, and enjoying the gift in commensality. Women who cohabited with someone continued cooking with joy and a sense of duty, as they have done before retirement, as long as they had the energy for it. For widows, especially those who had recently lost their spouse, the whole meaning of cooking and eating was lost and they were at risk of poor nutritional intake [21].

The challenge is to enable older people to be active despite any health limitations, if it is their preference to be socially engaged. The growing proportion of older adults who are dependent is of great concern for the sustainability for countries’ social welfare systems and participating in health-promoting activities can contribute to improved health outcomes [35].

## 2. A Proposed Theoretical Model for Sustainable and Safe Commensality Among Older Adults

Participating in health-promoting activities can contribute to improved health outcomes and improved social interaction among older adults. The proposed model (Figure 1) can serve as a conceptual framework when implementing FCOA in communities and research. There are three main core features in the model: (1) FCOA, contributing with education on healthy eating, cooking skills, and food safety, (2) Organized networking, including self-efficacy for simplified food procurement, food safety and trust, and social interactions, (3) Sustainable commensality, where outcomes such as the frequency of commensality, health effects, and well-being will be investigated.

### 2.1. Food Classes for Older Adults

A targeted measure to promote active aging could take the form of a community-organized activity like FCOA [36]. FCOA provides education on the core issues—healthy eating, cooking skills and food safety—as described in Table 1. FCOA is based on five group sessions. Each session lasts approximately three hours and follows an outline with an introduction and a theoretical part on nutrition, cooking, and food safety. Every session should also comprise a cooking part, commensality, and a summary. During the cooking part, each participant follows a recipe, preparing and cooking two portions of a complete meal: one to eat together with other participants during the session and one to take home. To compare home-cooked dishes with similar readymade meals, sensory testing/evaluation and discussions should be incorporated, covering nutrient content, price, food safety, and sensory aspects (Figure 1). Participating in FCOA may improve knowledge regarding healthy food choices, cooking, and safe food handling.

In 2007–2012, FCOA was piloted as a cooperative activity between the municipality of Uppsala and Uppsala University [36]. A questionnaire (*n* = 209) was distributed after the pilot. The results showed that 40 percent of the participants signed up for the food classes due to a need to learn more about food preparation in general, with a significantly higher proportion of men (51%) than women (33%, *p* < 0.05) [36]. Compared with women, significantly more men reported that they had received new information regarding food handling during FCOA. Furthermore, only cohabiting men reported that the reason for attending FCOA was a need to be prepared for future health changes in the household. Cohabiting people, both men and women, signed up for FCOA to a greater extent (48%) than single people (33%, *p* < 0.05), stating that they wanted to learn about food preparation in general. Almost 50 percent indicated that they had learned about healthy eating. A majority—91 percent of the participants—agreed with the statement *I have received enough information to be able to manage the food situation in the future, even when my health is not so good* [36]. Almost all participants (99%) found sharing a meal with others during the course to be a positive experience, and about 10 percent indicated that they had made new friends who they met with after the course.

### 2.2. Healthy Eating and Cooking Skills

Participation in FCOA provides participants with tools for procuring food in a healthy way (Figure 1). The theoretical model proposed in this paper also highlights tools for procuring healthy food. The social activity in FCOA may promote health. Further, most past studies have focused on women and how they perceive cooking and eating meals when their life situation is changed [21]. However, limited research has focused on older men in a changed situation, where they have to take responsibility for their own food procurement. Johannesson et al. [22] studied gender differences in older adults regarding practices, knowledge, and attitudes regarding food habits and meal patterns [22]. Also, in their study, it was shown that women took greater responsibility for the household, while a large proportion of men saw cooking as something they were not able to do.

### 2.3. Food Safety

The theoretical model presented in Figure 1 emphasizes the importance of food safety. To adopt good hygiene practices in the home setting, consumers need to be informed about safety procedures related to domestic food handling, storage, and preparation. The home environment represents an important site for the spread of the pathogens responsible for foodborne diseases [24]. Older adults may have a weak immune defense, making knowledge of food hygiene important. Food handling comprises few but important rules such as washing hands, cold food storage, optimal heating temperatures and avoiding cross-contamination. Introducing simple food handling rules can be enabling; an example is the concept of “The four Cs”: *Cooking*, *Cleaning*, *Chilling,* and avoiding *Cross-contamination* [37]. During each FCOA session, the food safety principles are highlighted in the theory and integrated into practice (Figure 1).

## 3. Organized Networking

The second step in the theoretical model shows the next step, after FCOA (Figure 1). Organized networking should be regarded as “filling a gap” for home-dwelling older adults, which could lead to new contacts and friends. Earlier studies have shown that the positive socialization effect of eating is dependent on friendship among those who eat together, i.e., *co-action effects* [5]. From a research perspective, baseline data can be collected and follow-up data at the last session. Parameters that measure quality of life should be central. Depending on the focus of a future research project, data that measure food and nutrition intake over time could also be collected. The FCOA concept has the potential to be expanded to organized networking. This could promote self-efficacy for simplified food procurement, food safety and trust, and social interactions.

### 3.1. Self-Efficacy for Simplified Food Procurement

*Self-efficacy* could be interpreted as an individual’s sense of control or belief in their own ability to change a behavior [38]. Richert et al. [39] examined how confidence in their own ability, or *self-efficacy*, affected people’s success in changing their diets. The authors concluded that a person’s self-confidence played a major role when they converted intentions into action, for example, increasing their intake of fruits and vegetables. People with low self-confidence needed to be encouraged to believe in their abilities before they could take in information and embrace healthy habits [39]. With a “green card” in cooking and food handling, self-confidence and safety could be increased. A structured “team” concept could be used to encourage FCOA participants to organize and plan a series of at least five meals, with cooking responsibility alternating between them. Validated instruments should be used to investigate self-efficacy.

### 3.2. Food Safety and Trust

The term “trust” has been used in the sense of the individual and subjective response to the objective and rather abstract notion of “risk” [40]. Food safety trust refers to a feeling that—in contrast to risk—cannot be calculated and that is related to a consumer’s attitude. Consumers have been shown to be divided in four categories dealing with trust in different ways: the naïve, the sensible, the skeptical and the denying consumers [40]. Ozawa and Sripad [41] identified core areas when measuring trust in health systems: communication, honesty, confidence, competence, fidelity, system trust, and fairness.

Consumers’ food safety trust is situational and a crucial question is if consumers are inclined to trust a very good friend who makes a meal to a greater extent than they do a restaurant. In a qualitative analysis of a European population, it was shown that men and women expressed somewhat differing worries and limitations regarding how to handle food. Men expressed no or only limited experience of food procurement and cooking, while the women expressed concerns about how to eat well later in life, when they might have limited strength to cook using raw ingredients [42]. Generally, there is a need to minimize the risk for intake of infected foods in private homes [24]. Food safety knowledge is required to safeguard against all risks—from procurement to consumption, i.e., transport, storage, and cooking (Figure 1). Creating a commensality network would without doubt require a kind of “food safety calibration,” so that all individuals involved would have the same level of food safety knowledge and share the same positive attitude towards food safety as an important issue.

## 4. Social Interactions

Socialization in general, and sharing meals in particular, is judged to be an important factor for eating. Good health also has a high impact on older people’s life satisfaction [43]. The expected outcome of organized networking and FCOA is that commensality will begin to occur spontaneously in the participants’ home environments (Figure 1). At least two people are needed to achieve sustainable commensality.

## 5. Sustainable Commensality and Proposed Measurements

The third and final step in the theoretical model is to investigate sustainable commensality (Figure 1). The outcomes can be measured by investigating the frequency of commensality, health effects, and well-being (Figure 1). In an interdisciplinary approach, various kinds of methods are proposed. A new instrument including quantitative and qualitative methods should be developed for use in future research. Possible relationships between commensality frequency and other effects on health and well-being should be evaluated, as suggested above.

### 5.1. Commensality Frequency

Through a questionnaire, data on the commensality frequency, i.e., how many times per week or month a person is eating with others at the same table, can be collected. The collection of data should be performed at the first FCOA session and at the end of the organized networking (Figure 1).

### 5.2. Health Effects

In a review by Vesnaver and Keller [4], it was concluded that social activity is an important factor for improved diet. However, more studies, especially longitudinal ones, were required to understand how different social factors influenced diet.

During the first FCOA session, some important variables regarding health status can be collected (Figure 1). Further, the well-being status of each participant can be determined. Medical variables—such as body weight, blood pressure, blood lipids, nutritional status, and frequency of foodborne infections—are suggested for collection. Well-being should be investigated through use of qualitative methods such as focus groups or qualitative interviews. The same parameters would also be collected at least one year after FCOA—but to study sustainability, even longer follow-up is needed.

### 5.3. Well-Being

Social isolation is an important risk factor predicting poor health in older adults [4,14]. Procuring meals for the family, relatives, and friends has been revealed to be an essential part of older women’s feeling of mental well-being [21,44]. Sharing meals with others, good health, and food knowledge were considered important sources of satisfaction with food-related life [45]. As people sign up for FCOA voluntarily, it is our hypothesis that the organized network will create a certain level of food enjoyment (Figure 1). When cooking tasty food that is eaten at a table in a group with people who know and trust each other, there might be a commensality effect (Figure 1). Herman identified *co-action effects* and also suggested some situational effects on meal intake [5]. It was found that eating with family and friends led to greater intake than eating with strangers. Hetherington [16] concluded that future research should address the importance of attentional processes in the development of satiation and satiety and that eating with friends and family required careful planning to prevent social facilitation effects. Marovelli [46] referred to a successful experiment among people living in London who prepared and shared meals. The distance between those who prepared food and those who consumed it was shortened, as the participants were involved in both food preparation and eating. The commensality effect created an atmosphere that fostered the sharing of experiences, with some variables considered unmeasurable [46].

In a report from the Swedish National Institute of Public Health, it was concluded that togetherness and participation in social activities are important for health and well-being [47]. In the suggested evaluation of FCOA, individual qualitative interviews covering each participant’s description of how they feel would be performed at the start of FCOA and at the end of the organized networking (Figure 1). Each participant would be asked to describe their situation related to well-being before and after the commensality networking. The results could contribute with new insights into how older adults experience these activities longitudinally.

As concluded by Sahyoun et al. [48], we need to learn more about how to achieve sustainable changes and study the consequences of these changes over the long term. Nutrition education interventions should be set within a social and environmental context [48]. From an international perspective, it is also important to pay attention to cultural differences. Thus, the theoretical model presented in this article should be modified based on all the aforementioned perspectives. The suggested model could then be applied when designing community health interventions [49].

## 6. Conclusions

In this article, a theoretical model for sustainable commensality among older adults has been proposed. The main aim of activities like FCOA is to enable the older population to acquire knowledge about cooking, eating, and food safety, to keep them healthy and socially active. The proposed theoretical model for sustainable and safe commensality among older adults can serve as a framework for future research. The situational effects of commensality on meal intake and to what extent the presence and behavior of others inhibit or disinhibit eating can be analyzed. The critical issue is the organized networking initiated by the food class practitioners, which is to be continued over a period of change. Therefore, efforts for sustainability would be the key issue determining whether the effects of commensality contribute to maintaining or improving health. The results of such studies could contribute with new insights into how older adults longitudinally experience the community-organized interventions.

## Figures and Tables

**Table 1:** The comprehensive manual for “Food Classes for Older Adults” [36].

**Session 1 (3 h)**	**Nutritious food—how do I know it is good enough to promote good health?**
	- What is healthy food?
	- What is special about food for older adults? Is there anything special about it?
	- Why is there a risk simply from being “old”? Signals to consider before losing weight?
	- How do I know if I am eating well? Choosing the “right food” in the “right portion sizes” for me. Using the “Plate Model” for a varied diet.
	- Keeping a well-established pattern of food choices.
	**Practical and social part**
	**Cooking and commensality**
	**Summing up**
**Session 2 (3 h)**	**Taste, smell, and hygiene. How to handle food to avoid ill health**
	- Taste, smell, and eating.
	- Food hygiene—the importance of handling food in the right way.
	- Food to be stored in the freezer.
	- Food to be stored at room temperature.
	**Practical and social part**
	**Cooking and commensality**
	**Summing up**
**Session 3 (3 h)**	**Practical aspects—food ingredients and food procurement**
	- Read the labels.
	- Read information on energy and nutrients.
	- Food shopping, practical issues.
	- Simple cooking when you don’t have the strength.
	**Practical and social part**
	**Cooking and commensality**
	**Summing up**
**Session 4 (3 h)**	**To cook or not to cook**
	- Processed food—a solution when you do not have the strength to cook.
	- Choosing food in the “jungle” of industrial/readymade meals. What’s good and what’s not good for my health?
	- Easy cooking without too much experience. Microwave cooking/one-pot meals.
	- Basic cooking—preparing meals for one, two or many meals?
	- Social aspects—meals with others?
	- How to find recipes, using the internet.
	**Practical and social part**
	**Cooking and commensality**
	**Summing up**
**Session 5 (3 h)**	**The contents of the last session are based on the participants’ requests**
	**Theory part**
	**Practical and social part Cooking and commensality**
	**Summing up**
